# The Health of India’s Elderly Population: A Comparative Assessment Using Subjective and Objective Health Outcomes

**DOI:** 10.1007/s12062-015-9122-2

**Published:** 2015-07-24

**Authors:** Jane Murray Cramm, Lisa Bornscheuer, Anna Selivanova, Jinkook Lee

**Affiliations:** Department of Health Policy & Management (iBMG), Erasmus University Rotterdam, Oudlaan 50, 3062 PA Rotterdam, The Netherlands; Center for Economic & Social Research, University of Southern California, 635 Downey Way, Suite 312, Los Angeles, CA 90089-3332 USA

**Keywords:** Grip strength, Lung function, Self-rated health, Activities of daily living, Elderly, India

## Abstract

This study examined relationships between and predictors of objective and subjective health measures among 766 individuals aged ≥ 45 years in India using the 2010 pilot wave of the Longitudinal Aging Study in India (LASI). Correlations between and gender differences in objective [grip strength, lung function] and subjective [self-rated health (SRH), dependence in activities of daily living (dADL)] health measures were examined. Multivariate logistic regression analyses, accounting for sample design, were conducted to identify predictors of poor health. Fewer individuals were classified as at risk according to subjective (SRH, 9 %; dADL, 12 %) than objective (lung function, 57 %; grip strength, 77 % women, 87 % men) indicators. Poor SRH was only weakly correlated with dADL (*r* = 0.103, *p* ≤ 0.05) and grip strength (*r* = −0.138, *p* ≤ 0.001). From this study we conclude that older Indians tend to report more positive perception of health than the objective measures of health indicates, and that subjective and objective health indicators capture different aspects of health and only weakly correlated.

## Introduction

The global demographic transition that we are witnessing is caused by simultaneous trends of declining fertility rates coupled with longer life expectancies. Where economic development – which is closely linked to these trends via channels such as advancement in medical technologies – is particularly rapid, so is population ageing (Chan 2005; Singh et al. 2013). India falls under the United Nations’ definition of ‘ageing’ countries^1^ and is a prime example of a context in which morbidity and mortality patterns are changing rapidly (Bhat and Dhruvarajan 2001; Prakash 1999). India’s elderly population has grown in relative and absolute terms (Bhat and Dhruvarajan 2001; Prakash 1999), and the United Nations Population Division projects that India’s population ages 50 and older will reach 34 % by 2050 (UN 2001). This demographic transition has important social, economic, and public health implications; one example is increased dependency ratios (Bhat and Dhruvarajan 2001; Chan 2005; Fikree and Pasha 2004).

Some of these implications are direct consequences of the epidemiological transition that takes place concurrently. India’s changing health burden is characterised by the increased importance of non-communicable diseases (e.g., chronic illnesses) and social and behavioural disorders, relative to more traditional areas of health intervention that are focused on infectious diseases (Chan 2005; Johnson 2005). Efficient public health decisions require a thorough understanding of these developments, and the examination of health outcomes in ageing populations is one way of fostering this understanding. Against this background, this study aims to add to the literature examining the usefulness of different health indicators, with particular regard to self-rated and objective health measures.

## Literature Review

A variety of measures have been used to examine individuals’ health status. The most frequently used measures are subjective ones, such as self-rated health (SRH) and self-perceived dependence in activities of daily living (ADL), and also recently few objective measures such as grip strength and lung function gained popularity (Ziebarth 2010). SRH and grip strength are static indicators, capturing deviations from a norm perceived as healthy (Murray and Chen 1992; Ziebarth 2010).

By contrast, dependence in ADL is a functional indicator, capturing the inability to perform certain tasks. Dependence in ADL has been associated with various outcomes of interest, such as admission to retirement homes, health care utilisation, and mortality (Tsuji et al. 1994; Luppa et al. 2010; Scott et al. 1997). The measure is particularly useful for assessment of the health status of elderly populations, where prevalence rates are especially high (Wiener et al. 1990). Experience is lacking, however, in its use in the context of developing countries, such as India (Fillenbaum et al. 1999).

Grip strength has been associated with a variety of health outcomes and can be used as a proxy for overall muscle strength. Low grip strength has been found to be a consistent predictor of mobility limitations and all-cause mortality (Bohannon 2008; Metter et al. 2002; Rantanen et al. 2000; Sallinen et al. 2010). Lung function has also been found to be a predictor of all-cause mortality in both sexes and different age groups, even when adjusting for factors such as body mass index or smoking (Hole et al. 1996; Knuiman et al. 1999; Mannino et al. 2003; Neas and Schwartz 1998; Schunemann et al. 2000).

Several studies have found that subjective health measures are independent predictors of a variety of health outcomes and can thus be said to predict different ‘parts’ of mortality than more conventional, objective measures (Idler and Benyamini 1997). SRH, among the most frequently used measures, is an ardently debated indicator (Idler and Benyamini 1997; Johnston et al. 2009; Smith and Goldman 2011). SRH has repeatedly been proven to be a powerful and independent predictor of diverse health outcomes, and a stronger predictor of mortality than physician-assessed health as evident by a review of twenty-seven international community studies (Idler and Benyamini 1997) and empirical evidence from developed countries (Alexopoulos and Geitona 2009; Deeg and Bath 2003; Eriksson et al. 2001; McCullough and Laurenceau 2004). Even researchers who could not confirm the independent character of this predictive power, such as Bath (2003) in his study on the relationship between SRH and mortality in elderly men and women concede that differences in study design and cross-cultural variation may underlie the inconsistency of findings (Bath 2003). However, variations in the predictive ability of SRH have been found with regard to age, gender, and length of follow up (Bath 2003; Idler 2003; Jylhä 2009); for example, SRH seems to lose some predictive power in the assessment of older women’s health status (Benyamini et al. 2000; Cooper et al. 2010; McCullough and Laurenceau 2004). The origin of these variations is not clear (Smith and Goldman 2011; Spiers et al. 2003); several possible explanations have been proposed, such as the greater sensitivity of SRH to certain health conditions with differing prevalence across genders (Deeg and Bath 2003; Deeg and Kriegsman 2003). Reporting bias is another issue that complicates the use of SRH (Bago d'Uva et al. 2008). For example, Lindeboom and van Doorslaer (2004) confirmed the occurrence of index and cut-point shifting for age and gender when testing for differential reporting in subjective measures such as the McMaster Health Utility Index. If the variations observed in response patterns to health surveys are systematic and related to socio-demographic differences – as they likely are – they severely hinder the correct assessment of health (Bago d’Uva et al. 2008; Sen 2002). Therefore, the state of the art allows us only to state that SRH ‘clearly measure[s] something more – and something less – than objective medical ratings’ (Maddox and Douglass 1973; p. 92).

Amartya Sen (1993, 2002) calls for further caution when using SRH in India. Sen argues that the individual’s perception of health might strongly be shaped by the respective socio-economic context. Particularly, where there is an overall lack of (health) literacy - often the case in areas of poor socio-economic standing - measures of SRH might not be reliable because individuals simply fail to acknowledge certain morbidities, but perceive them to be normal. Sen’s hypotheses are supported by studies such as the one conducted by Sudha et al. (2007) in South India in an elderly population, finding that marital status may bias measures of SRH, and may do so differently across genders.

Holding against these concerns is e.g., a study conducted by Subramanian et al. (2009), that maintains that those individuals with lower SES are in fact more likely to report morbidities, even when controlling for the objective level of health. Supporting the validity of self-rated health measures are also findings from a study amongst people of 50 years or older in Bangladesh, where SRH was found to be significantly associated with measured physical performance, even when controlling for other factors (Rahman and Barsky 2003). Furthermore, Hirve et al. (2012) found a significant association between SRH and death independent of SES for an elderly Indian population segment. However, this study again also points to potential differences in SRH validity across genders.

The use of subjective health measures is the norm in surveys; objective measures and biomarkers are rarely used in this context. The introduction of such measures may provide an important complement to SRH and similar indicators (Ambrasat et al. 2011; Johnston et al. 2009; Kakwani et al. 1997). Thus, this study aimed to increase our understanding of health among elderly individuals in India by investigating relationships between subjective (SRH and dependence in ADL) and objective (grip strength and lung function) health indicators, and examining variation in predictor variables among health outcomes.

## Methods

This study employed cross-sectional data from the 2010 pilot wave of the Longitudinal Aging Study in India (LASI), conducted in collaboration by the Harvard School of Public Health, the International Institute for Population Sciences, and the RAND Corporation. The primary objective of LASI is to provide Indian policymakers with the information needed to improve health status and health behaviour in the country’s ageing population. Social, economic, and health data (e.g., income, work/employment history, SRH, blood pressure, grip strength) were collected from a nationally representative sample of the population aged ≥ 45 years.

### Sample

The LASI pilot study took place in the northern Indian states of Rajasthan and Punjab, and the southern states of Kerala and Karnataka. The sampling plan was based on the 2001 Indian census. To adequately represent different socio-economic conditions, the eight districts that served as primary sampling units were stratified across urban and rural areas. Eligible households had at least one member aged ≥ 45 years, and eligible individuals were aged ≥ 45 years or married to an individual of that age. A total of 1546 households was randomly sampled, and 1683 interviews were conducted in 950 of these households. The response rates were 88.5 % for households and 90.9 % for individuals. Within the bounds of the individual survey, grip strength, lung function, and other biomarker data were collected from a smaller sample of 928 consenting eligible individuals (interviewees and their spouses aged ≥ 45 years) (Chien et al. 2013a, b, 2014).

### Outcome Measures

#### Self-Rated Health

SRH was assessed by asking respondents to rate their current overall health on a five-point ordinal scale ranging from poor (1) to excellent (5) (full range: poor, fair, good, very good, excellent). This outcome variable was dichotomised as poor (1, 2) or good (3–5) (Chien et al. 2013a, b; Cramm and Lee 2014).

#### Dependence in Activities of Daily Living

Respondents’ ability to perform ADL was assessed using six variables: walking across the room, dressing oneself, bathing oneself, eating, getting out of bed, and using the toilet. Each item had four possible responses indicating that the respondent had difficulty (1) or no difficulty (2) with the task, or could not (3) or did not want to (4) perform the task. Responses 1 and 3 were considered to indicate some difficulty (coded as 1), 2 was considered to indicate no difficulty (coded as 0), and responses of 4 were recorded as missing values.

The LASI team derived two summary ADL measures. The first measure used the ADL recommended by Wallace and Herzog (1995), including ‘some difficulty’ scores for the individual measures of bathing, dressing, and eating. The second summary measure additionally included getting in/out of bed and walking across the room. Summary measures were computed only for respondents with no missing value for any individual measure (Chien et al. 2013a, b). The summary variable was dichotomised as difficulty or no dependence in ADL.

#### Grip Strength

The LASI team measured grip strength using the sex-specific cut-off points reported by Sallinen and colleagues (2010): 21 kg for women (67 % sensitivity, 73 % specificity) and 37 kg for men (62 % sensitivity, 76 % specificity) (Chien et al. 2013a, b, 2014). This outcome measure was dichotomised as at risk (below cut-off value) or not at risk (cut-off value or above).

#### Lung Function

The LASI team used spirometry to measure lung function by examining the amount (volume) and speed (flow) of air that an individual was able to inhale or exhale. Measurement took place in the population setting. The flow electronic volume [forced expiratory volume in 1 s (FEV_1_) / forced vital capacity (FVC)] was used to identify lung problems. The criteria advanced by the Global Initiative for Chronic Obstructive Lung Disease (GOLD) were used to classify the severity of chronic obstructive pulmonary disease (COPD, defined as FEV_1_ / FVC < 0.70). The severity of airflow limitation in COPD, was characterised as mild, moderate, severe, or very severe) (Chien et al. 2013a, b, 2014; Soriano et al. 2013). This health measure was dichotomised as normal or abnormal spirometry values.

#### Socio-Demographic Measures

The socio-demographic measures used in this research are age, marital status, educational attainment, quintiles of income, place of residence, caste, and gender. Place of residence was categorised in terms of rural/urban environment. Educational status was classified using four categories (illiterate, primary, secondary and higher education). Castes were classified as scheduled castes, scheduled tribes, other backward group (OBG) and non-scheduled castes or tribes. The first two categories are historically disadvantaged social classes in India (Chien et al. 2013a, b). Furthermore, the variable smoking was included in the analysis. In addition, we constructed the variable ‘unhealthy environment’ which takes value of 1 if there is indoor smoking at home and/or the cooking is made under chimney, the variable takes value of 0 if there is no indoor smoking and cooking is not fulfilled under chimney. In such a way it is possible to observe if lung function is associated with smoking or unhealthy smoking environment at home.

### Statistical Analyses

Descriptive analyses were used to examine objective and subjective health measures. We corrected health characteristics for age-gender variations, so we used age-gender standardized values. Since we use data for elderly population (age 45 and more), age variable was split into 3 categories: age 45–60, age 60–75 and age>75. In addition, we used concentration curves to investigate the association between socioeconomic status and health outcomes (objective and subjective). Pearson’s product–moment correlation analyses were then used to investigate relationships between these measures. Differences in health outcomes between men and women were examined using chi-squared and independent-samples *t*-tests. Multivariate logistic regression analyses were used to identify and compare predictors of poor health across genders in India’s elderly population, while accounting for sample design effect.

## Results

Descriptive analyses showed that the relative distributions of individual health outcomes differed between subjective and objective measures. Although 9 % of respondents reported poor health and 12 % reported difficulties in ADL, nearly 57 % of respondents had below-normal spirometry values and the majority of respondents of both sexes (77 % of women, 87 % of men) had below-threshold grip strength values.

Tables 1 and 2 shows the descriptive statistics of health outcomes across socio-demographic characteristics among older men women in India (standardized).

**Table 1:** Descriptive statistics of health outcomes across socio-demographic characteristics among older men in India(standardized)

Over	SRH (poor) *n* = 712 Mean prevalence(SD)	Lung function (abnormal) *n* = 712 Mean prevalence(SD)	ADL (difficulty) *n* = 638 Mean prevalence(SD)	Grip strength (low) *n* = 712 Mean(SD)
Age				
45–60	7.4 % (4 % – 10.8 %)	56.8 % (49 % – 64.7 %)	7.1 % (4 % –10.2 %)	73.7 % (68.2 % – 79.3 %)
60–75	17.2 %(11 % – 23.4 %)	51.3 % (41.3 % –61.3 %)	16.6 % (10.3 % –23 %)	78.7 % (72 % –85.4 %)
Older than 75	22.5 % (10.6 % – 34.5 %)	38.3 % (23.9 % – 52.8 %)	25.7 % (12.9 % – 38.5 %)	80.1 %(68.15 % –92.1 %)
Education level				
Illiterate	11.1 % (6.8 % – 15.4 %)	49.7 % (42.4 % – 57 %)	11.6 % (7.7 % – 15.4 %)	78.1 % (73.5 % – 82.7 %)
Primary school	16.7 % (10.9 % –22.4 %)	70 % (61.8 % – 78.2 %)	13.9 % (6.3 % – 21.4 %)	68.3 % (59.6 % – 77.1 %)
Secondary school	13.1 % (6.6 % – 19.7 %)	69.1 % (60 % – 78.2 %)	10 % (6 % – 14 %)	73.1 % (65.3 % – 81 %)
Higher education	6.7 % (1.6 % – 11.7 %)	66.7 % (54.7 % – 78.6 %)	13.4 % (6.7 % – 20.2 %)	73.3 %(64.5 % –82.2 %)
Income quintiles				
1	14.5 % (7.5 % – 21.4 %)	14.1 % (6.5 % – 21.7 %)	56 % (43.4 % – 68.5 %)	78.9 % (71 % – 86.9 %)
2	11.3 % (5.7 % – 17 %)	11 % (5.5 % – 16.5 %)	56.7 % (42.3 % – 71.2 %)	87.3 % (80.6 % – 93.9 %)
3	13 % (6.1 % – 20 %)	12 % (4.9 % – 19 %)	75.3 % (67.9 % – 82.7 %)	92.2 % (87.7 % – 96.7 %)
4	10.8 % (4.8 % – 16.7 %)	8.3 % (2.7 % – 14 %)	52.7 % (42.6 % – 62.8 %)	84.4 % (76.8 % – 91.9 %)
5	11.2 % (4.1 % – 18.3 %)	11.3 % (5 % – 17.5 %)	64.6 % (47.9 % – 81.3 %)	83.7 % (75.4 % – 92 %)
Background				
Scheduled caste	13.6 % (5.2 %–21.9 %)	60.2 % (50.8 % – 69.6 %)	11.2 % (5.4 % – 17 %)	72.9 % (65.4 % – 80.4 %)
Scheduled tribe	2.9 % (2.5 % – 8.2 %)	77.1 % (61.5 % – 92.8 %)	0	82.9 % (69.4 % – 96.3 %)
Other background class	11.6 % (7.4 % – 15.8 %)	63.8 % (56.3 % – 71.2 %)	14.8 % (10.5 %–19.1 %)	77.5 % (72.4 % – 82.7 %)
None	13.3 % (8.5 % – 18 %)	51.2 % (43.7 % – 58.7 %)	10.4 % (6.3 % – 14.4 %)	71.9 % (65.9 % – 77.8 %)

**Table 2:** Descriptive statistics of health outcomes across socio-demographic characteristics among older women in India(standardized)

Over	SRH (poor) *n* = 723 Mean prevalence(SD)	Lung function (abnormal) *n* = 726 Mean prevalence(SD)	ADL (difficulty) *n* = 662 Mean prevalence(SD)	Grip strength (low) *n* = 725 Mean prevalence (SD)
Age				
45–60	10.1 % (6.2 % – 14 %)	57.7 % (51 % – 64.4 %)	10.9 % (6.5 % – 15.4 %)	73.8 % (68.8 % – 78.8 %)
60–75	19.8 % (13.3 % – 26.2 %)	44.4 % (37.8 % – 51.9 %)	18.3 % (12.3 % – 24.2 %)	69.9 % (63.4 % – 76.5 %)
Older than 75	38 % (24.2 % – 52 %)	37.1 % (23.5 % –50.8 %)	36.4 % (22.2 % – 50.6 %)	70.1 % (56.5 % – 83.8 %)
Education level				
Illiterate	16.7 % (12.4 % – 21 %)	48.2 % (42.6 % – 53.8 %)	15.6 % (11 % – 20 %)	73.2 % (68.9 % – 77.4 %)
Primary school	19.2 % (8.9 % – 29.5 %)	64 % (52.9 % – 75.1 %)	23.9 % (13.9 % – 33.8 %)	52 % (42.2 % – 61.8 %)
Secondary school	15.6 % (10.3 % – 21 %)	64.2 % (57.3 % – 71.1 %)	11.6 % (0.078 – 0.154)	70.9 % (61.8 % – 80.1 %)
Higher education	6 % (2 % –14.2 %)	75.8 % (60.4 % – 91.1 %)	9.38 % (0.2 % – 19 %)	51.5 % (29.8 % – 73.2 %)
Income quintiles				
1	11.7 % (4.7 % – 18.8 %)	47.6 % (34 % – 61.1 %)	14 % (6.4 % – 21.7 %)	80.6 % (73.9 % – 87.3 %)
2	13 % (7.5 % – 18.6 %)	56.3 % (44 % – 68.5 %)	16.9 % (10.4 % – 23.4 %)	80.3 % (75.2 % – 85.4 %)
3	11.1 % (6 % – 16.3 %)	61.1 % (53 % – 69.2 %)	11.7 % (6 % – 17.4 %)	82.5 % (75.6 % – 89.5 %)
4	26.4 % (16.9 % – 36 %)	52.7 % (42.7 % – 62.7 %)	22 % (11.8 % – 32.2 %)	68.7 % (59.4 % – 78 %)
5	14.3 % (7.6 % – 20.9 %)	56 % (43 % – 69.2 %)	11.9 % (5.3 % – 18.6 %)	73.8 % (63.5 % – 84.2 %)
Background				
Scheduled caste	11.5 % (5.9 %–17.1 %)	56.6 % (46.3 %–66.8 %)	15.3 % (8 %–22.6 %)	67.2 % (59.3 %–75.1 %)
Scheduled tribe	19 % (8.5 % – 29.6 %)	50 % (26.9 % – 73.1 %)	17.5 % (5.3 % – 29.7 %)	81 % (72.6 % – 89.3 %)
Other background class	15.8 % (10.9 % – 20.7 %)	57 % (50.8 % – 63.2 %)	11.5 % (7.9 % – 15.1 %)	66.2 % (61.5 % – 70.9 %)
None	19.8 % (13.5 % – 26.1 %)	52.2 % (42.9 % – 61.5 %)	20.6 % (14.3 % – 26.9 %)	69.9 % (63.3 % – 76.5 %)

**Graph 1:**
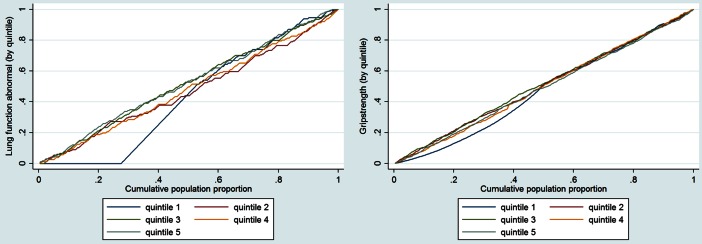
Cumulative population proportions

Concentration curves reveal a relationship between income and health outcomes (objective and subjective) (see Graph 1).

Objective and subjective health measures showed no correlation or weak relationships. Poor health was significantly correlated with dependence in ADL (*r* = 0.103, *p* ≤ 0.05) and grip strength (*r* = −0.138, *p* ≤ 0.001; Table 3).

**Table 3:** Pearson correlations between subjective and objective health outcomes

	1	2	3
1. Poor health (subjective)			
2. Grip strength (objective)	−0.138***		
3. Dependence in ADL (subjective)	0.103*	−0.049	
4. Abnormal spirometry value (objective)	0.052	0.052	−0.011

*ADL* activities of daily living. *p ≤ 0.05, ***p ≤ 0.001

Tables 4 and 5 present the results of multivariate logistic regression analysis of subjective and objective health indicators among men and women, respectively.

**Table 4:** Relationships between socio-demographic characteristics and health outcomes among older men in India

	SRH (poor) *n* = 640 Adjusted OR	Lung function (abnormal) *n* = 640 Adjusted OR	ADL (difficulty) *n* = 637 Adjusted OR	Grip strength (low) *n* = 640 Adjusted OR
Residence(rural)	1.341 (0.545 – 3.3)	0.666 (0.328 – 1.352)	0.819 (0.367 – 1.826)	0.683 (0.323 – 1.446)
Age(45–60)	0.199(0.076 – 0.519)^***^	3.189 (1.419 – 7.168) ^***^	0.112 (0.04 – 0.318)^***^	0.659 (0.24 – 1.809)
Age(60–75)	0.518 (0.213 – 1.262)	1.698 (0.744 – 3.875)	0.332 (0.125 – 0.885) ^*^	0.673 (0.219 – 2.066)
Education(primary)	1.28 (0.675 – 2.426)	3.865 (2.02 – 7.397) ^***^	1.045 (0.403 – 2.71)	0.368 (0.199 – 0.68) ^***^
Education(secondary)	1.146 (0.513 – 2.558)	3.02 (1.672 – 5.452) ^***^	0.774 (0.356 – 1.684)	0.434 (0.248 – 0.758) ^***^
Education(higher)	0.503 (0.176 – 1.439)	2.308 (1.378 – 3.868)^***^	1.028 (0.403 – 2.62)	0.513 (0.25 – 1.05)**
Income quintile 2(upper middle)	0.75 (0.326 – 1.72)	0.968 (0.475 – 1.973)	0.607 (0.228 – 1.613)	2.14 (0.896 – 5.115)**
Income quintile 3(middle)	1.308 (0.573 – 2.986)	2.993 (1.487 – 6.026) ^***^	1.18 (0.408 – 3.414)	2.859 (1.223 – 6.682) ^*^
Income quintile 4(poor)	0.946 (0.333 – 2.689)	0.748 (0.389 – 1.439)	0.603 (0.216 – 1.675)	1.504 (0.75 – 3.014)
Income quintile 5(poorest)	0.95 (0.386 – 2.342)	1.354 (0.545 – 3.362)	0.996 (0.370 – 2.679)	1.74 (0.854 – 3.542)
Marital status(single)	2.746 (1.102 – 6.845)^*^	1.193 (0.664 – 2.144)	0.873 (0.308 – 2.476)	1.343 (0.467 – 3.862)
Scheduled tribe	1.28 (0.675 – 2.426)	2.228 (0.753 – 6.593)	1	0.9 (0.363 – 2.235)
Other background class	1.146 (0.513 – 2.558)	0.791 (0.4 – 1.566)	1.305 (0.561 – 3.034)	1.48 (0.755 – 2.901)
No tribe/caste/background class	0.503 (0.318 – 2.164)	0.521 (0.263 – 1.034)**	0.982 (0.405 – 2.381)	0.98(0.519 – 1.848)
Smoker(yes)	1.352 (0.782 – 2.337)	1.319 (0.734 – 2.371)	2.234 (0.966 – 5.168)^**^	1.68 (0.866 – 3.529)
Indoor smoking (yes)	1.165 (0.662 – 2.051)	0.932 (0.503 – 1.724)	0.735 (0.312 – 1.73)	0.585 (0.309 –1.108)**
Cooking under chimney(yes)	1.234 (0.644 – 2.365)	1.339 (0.848 – 2.113)	0.878 (0.467 – 1.653)	1.339 (0.764 – 2.347)
Model F	1.76^**^	3.64^***^	3.04^***^	2.39^***^

*SRH* self-rated health, *OR* odds ratio, *ADL* activities of daily living

^*^ *p* ≤ 0.05; ^**^ *p* ≤ 0.1; ^***^ *p* ≤ 0.001

**Table 5:** Relationships between socio-demographic characteristics and health outcomes among older women in India

	SRH (poor) *n* = 667 Adjusted OR	Lung function (abnormal) *n* = 668 Adjusted OR	ADL (difficulty) *n* = 661 Adjusted OR	Grip strength (low) *n* = 668 Adjusted OR
Residence(rural)	1.689 (0.849 – 3.362) ^**^	1.06 (0.604 – 1.86)	0.815 (0.396 – 1.678)	1.34 (0.888 – 2.046)
Age(45–60)	0.2 (0.082 – 0.489)^***^	1.817 (0.907 – 3.64) ^**^	0.197 (0.099 – 0.392)^***^	0.609 (0.208 – 1.784)
Age(60–75)	0.456 (0.187 – 1.113)^**^	1.145 (0.556 – 2.359)	0.297 (0.123 – 0.392) ^***^	0.634 (0.237 – 1.701)
Education(primary)	1.179 (0.603 – 2.305)	2.592 (1.513 – 4.442) ^***^	1.788 (0.881 – 3.626)	0.402 (0.222 – 0.727) ^***^
Education(secondary)	1.09 (0.538 – 2.205)	1.873 (1.196 – 2.933) ^***^	0.857 (0.46 – 1.596)	1.164 (0.65 – 2.086)
Education(higher)	0.433 (0.091 – 2.048)	3.571 (1.431 – 8.91) ^***^	0.653 (0.159 – 2.689)	0.425 (0.153 – 1.178) ^**^
Income quintile 2(upper middle)	0.956 (0.409 – 2.235)	1.366 (0.702 – 2.661)	1.086 (0.42 – 2.811)	1.001 (0.509 – 1.969)
Income quintile 3(middle)	0.85 (0.34 – 2.126)	1.852 (1.003 – 3.419) ^*^	0.703 (0.311 – 1.592)	1.079 (0.509 – 2.29)
Income quintile 4(poor)	2.421 (1.072 – 5.466) ^*^	1.254 (0.654 – 2.405)	1.461 (0.628 – 3.397)	0.471 (0.238 – 0.931)*
Income quintile 5(poorest)	1.093 (0.445 – 2.687)	1.17 (0.506 – 2.708)	0.873 (0.345 – 2.213)	0.728 (0.32 – 1.659)
Marital status(single)	2.971 (1.902 – 4.64) ^***^	0.8 (0.546 – 1.173)	2.492 (1.407 – 4.412) ^***^	1.369 (0.836 – 2.242)
Scheduled tribe	1.666 (0.648 – 4.28)	1.011 (0.293 – 3.494)	1.272 (0.454 – 3.567)	1.777 (0.578 – 5.47)
Other background class	1.735 (0.793 – 3.796)	1.13 (0.629 – 2.03)	0.881 (0.512 – 1.518)	0.872 (0.547 – 1.39)
No caste/tribe/background class	2.075(0.889 – 0.844)**	0.84 (0.427 – 1.65)	1.437 (0.737 – 2.8)	1.403 (0.856 – 2.3)
Smoker(yes)	1.175 (0.502 – 2.754)	1.292 (0.645 – 2.587)	2.39 (1.05 – 5.444)^**^	3.419 (0.807 – 14.486)^***^
Indoor smoking(yes)	1.846 (1.02 – 3.34)*	0.881 (0.495 – 1.567)	1.482 (0.787 – 2.789)	1.112 (0703–1.758)
Cooking under chimney(yes)	0.634 (0.357 – 1.127)	1.72 (1.092 – 2.709)*	0.688 (0.409 – 1.157)	0.644 (0.383 – 1.082)**
Model F	3.27^***^	1.96^*^	3.35^***^	2.22^*^

*SRH* self-rated health, *OR* odds ratio, *ADL* activities of daily living

^*^ *p* ≤ 0.05; ^**^ *p* ≤ 0.1; ^***^ *p* ≤ 0.001

Among men, older age and being single increased the probability of being in poor health; age, educational level, income and tribe/caste/background class predicted lung function; older age and smoking increase the likelihood of dependence in ADL; educational level, income and indoor smoking predicted grip strength.

Among women, residence, age, marital status, income and indoor smoking predicted SRH; age, educational level, cooking under chimney and income predicted lung function; age, income and smoking predicted ADL; educational level and smoking predicted grip strength.

## Discussion

This study added weight and detail to the findings of similar studies with the use of two objective health outcomes, as well as subjective measures, in the assessment of health in India’s elderly population. The most striking finding of this study is the weakness or lack of correlation between subjective and objective health indicators. These findings were also reported by Goverover and colleagues (2005) in their study investigating the relation between subjective and objective measures of everyday life activities in persons with multiple sclerosis. They concluded that all correlations between subjective and objective functional performance measures were low and non-significant, which is consistent with other studies in among patients with MS (Hoogervorst et al. 2003; Doble et al. 1994), Alzheimer patients (Bertrand and Willis 1999) and hospitalized older patients (Sager et al. 1992). The lack of a significant relation between objective and subjective health measures suggests that they each provide unique contributions to the evaluation of health status. Self-report can provide information about a person’s own perceptions regarding their health that cannot be measured using an objective assessment tool. Objective health measures are usually task oriented and are rated along a number of physical and/or cognitive dimensions enabling the observer to make a judgment as to a very specific aspect of health or health activity that can or cannot be performed independently (Gitlin 2001). As such, reliance solely on self-report health measures seem to provide information that may not reflect actual health performance (e.g., grip strength or lung function) in everyday life (Goverover et al. 2005). Longitudinal research indeed showed that perceived or subjective health is a significant predictor of both mortality and morbidity even more so than objective health measures (Menec et al. 1999; Mossey 1995; Mossey and Shapiro 1982; Reuben et al. 1992). Furthermore, two literature reviews (Idler and Benyamini 1997) proved that subjective health remained a significant predictor of mortality, after controlling for objective health status as well as other covariates such as socioeconomic status, social support, and risk behaviours. This calls for the inclusion of both subjective as well as objective health indicators since they seem to catch ‘different parts’ of a person’s overall health.

The limitations of this study include the cross-sectional nature of the data, which prevented the examination of causality. Furthermore, it would have been beneficial to include a measure of economic dependency in the analysis, similar to the approach taken by Roy and Chaudhuri (2008). While this study included both objective and subjective health indicators and shed some light on their (lack of) relationship there is still a lot to learn. While in line with earlier studies (Goverover et al. 2005; Hoogervorst et al. 2003; Bertrand and Willis 1999; Doble et al. 1994; Sager et al. 1992) we found that objective and subjective health indicators were not or only very weakly related to each other use of other health measures, however, might lead to different findings. Still, we believe that a strength of this study is the inclusion of subjective as well as objective indicators of health. Biomarker data collection is a valuable contribution of the LASI study, as such information – crucial for the assessment of health status – is scarce given India’s poor health care infrastructure (Chien et al. 2013a, b, 2014).

In conclusion, the main contribution of this research to the existing literature is the finding that no strong relationship between the health outcomes examined persists. The disparity between the small percentages of the population classified as ‘at risk’ according to subjective measures, and the majority of individuals determined to be at risk according to objective indicators, suggests that they reflect different ‘parts’ of mortality and morbidity. While ADL or SRH is expected to be low in our study sample the high proportion of older people in India with low grip strength and poor lung function is a concern. Further study is thus warranted to obtain a full understanding of the correct application and interpretation of different health indicators depending on the problem of interest, the context in which it is investigated, and the population involved.

## Footnotes

1 A country is defined as ‘ageing’ when the percentage of the population aged ≥ 60 years reaches 7 %.
